# Usability and Utility of a Mobile App to Deliver Health-Related Content to an Older Adult Population: Pilot Noncontrolled Quasi-Experimental Study

**DOI:** 10.2196/46151

**Published:** 2024-05-17

**Authors:** Marta Lemos, Ana Rita Henriques, David Gil Lopes, Nuno Mendonça, André Victorino, Andreia Costa, Miguel Arriaga, Maria João Gregório, Rute de Sousa, Helena Canhão, Ana M Rodrigues

**Affiliations:** 1 Unidade de Saúde Pública do ACES Algarve II – Barlavento Centro de Saúde de Portimão Portimão Portugal; 2 CHRC, NOVA Medical School NMS Universidade NOVA de Lisboa Lisboa Portugal; 3 Faculdade de Ciências e Tecnologia Universidade NOVA de Lisboa, Costa da Caparica Almada Portugal; 4 Direção-Geral de Saúde Lisboa Portugal; 5 Instituto de Saúde Ambiental Faculdade de Medicina Universidade de Lisboa Lisboa Portugal; 6 Nursing Research Innovation and Development Centre of Lisbon (CIDNUR) Nursing School of Lisbon (ESEL) Lisboa Portugal; 7 Catolica Research Centre for Psychological, Family and Social Wellbeing Lisboa Portugal; 8 Faculdade de Ciências da Nutrição e Alimentação Universidade do Porto Porto Portugal; 9 Programa Nacional para a Promoção da Alimentação Saudável Direção-Geral da Saúde Lisboa Portugal

**Keywords:** DigiAdherence, mHealth, mobile app, technology, utility, usability, ICT, application, patient-centered, tool, prevention, falls, treatment, nutrition, physical activity, pilot study, older adults, adherence, engagement, compliance

## Abstract

**Background:**

Digital patient-centered interventions may be important tools for improving and promoting social interaction, health, and well-being among older adults. In this regard, we developed a mobile app called DigiAdherence for an older adult population, which consisted of easy-to-access short videos and messages, to improve health-related knowledge among them and prevent common health conditions, such as falls, polypharmacy, treatment adherence, nutritional problems, and physical inactivity.

**Objective:**

This study aimed to assess the usability and utility of the DigiAdherence app among Portuguese older adults 65 years or older.

**Methods:**

In this pilot noncontrolled quasi-experimental study, older adults who were patients at the primary health care center in Portimão, Portugal, and owned a smartphone or tablet were recruited. Participants were assessed at baseline, given access to the DigiAdherence app for 1 month, and assessed again immediately after 30 days (first assessment) and 60 days after stopping the use of the app (second assessment). App usability and utility (primary outcomes) were analyzed in the first follow-up assessment using a structured questionnaire with 8 items. In the second follow-up assessment, our focus was on knowledge acquired through the app. Secondary outcomes such as treatment adherence and health-related quality of life were also assessed.

**Results:**

The study included 26 older adults. Most participants rated the different functionalities of the app positively and perceived the app as useful, attractive, and user-friendly (median score of 6 on a 7-point Likert scale). In addition, after follow-up, participants reported having a sense of security and greater knowledge in preventing falls (16/24, 67%) and managing therapies and polypharmacy (16/26, 62%).

**Conclusions:**

The DigiAdherence mobile app was useful and highly accepted by older adults, who developed more confidence regarding health-related knowledge.

**International Registered Report Identifier (IRRID):**

RR2-10.2196/29675

## Introduction

### Background

People worldwide are living longer. By 2030, 1 in 6 people in the world will be 60 years or older [1]. Portugal has one of the oldest populations in Europe due to an increased life expectancy and reduced fertility rate. In 2021, older adults comprised 22.4% of the population, a rapid increase of 3.7% from 18.7% in 2011 [2].

The World Health Organization defines health promotion as the process of enabling people to take increasing control over their health and its determinants, thereby improving their health [1], while health literacy encompasses a person’s ability to access, understand, appraise, and apply health information to make informed decisions regarding their health [3]. In light of this, it is crucial to highlight the findings from the Health Literacy Population Survey Project 2019-2021, which suggests a strategic focus on interventions that improve health literacy for health promotion, disease prevention, health care, and health system navigation. The results of this study also suggest a social gradient for health literacy; women, older age groups of individuals, people with lower economic capacity, people with lower levels of schooling, and the unemployed are at risk of low health literacy in Portugal. Therefore, particular attention should be given to this population regarding actions to promote health literacy [4].

As the world transitions into the information age, incorporating digital technologies into health promotion is becoming commonplace. In the past two decades, the use of digital media and digital products for health promotion has grown exponentially, particularly mobile app–based health promotion programs [5]. As the current generation of older adults is becoming more accustomed to using digital products [6], we must consider exploring digital patient-centered interventions as tools to improve and promote social interaction, encourage healthy lifestyles, and enhance well-being and quality of life (QoL) among this age group [7-9]. Evidence suggests that older adults with a higher education level and self-rated health status are more willing to use health care technologies [10-12]. Several research studies have been performed on mobile health (mHealth) apps, and the results suggest that, when well designed, these apps can improve health literacy skills, self-care, and treatment adherence; empower patients; and reduce the cost of health care [8,13-17]. We previously developed an informative, motivational, interactive, home-based TV application intervention program for an older adult population (Saúde.Come Senior) to promote healthy lifestyles [18]. This program has been fully implemented and tested, and shown to be well accepted and successful in reducing food insecurity and improving participants’ physical function [19].

As older adults may be potential users of mHealth apps, it is crucial to study, design, and adapt technologies to make them intuitively understandable for this age group [20-23]. This is challenging because the range of technical abilities of 65- to 95-year-olds is vast and very heterogeneous [20]. Because this demographic represents a diverse range of technical abilities and faces unique challenges related to aging that can impact their interaction with digital technology, addressing the usability and utility of mobile apps among older adults, along with users’ overall satisfaction and willingness to use them, is of paramount importance [8,24]. These challenges include cognitive changes, sensory limitations, and the need for technology that accommodates reduced dexterity and potential visual, hearing, and memory impairments [25-27]. Additionally, factors such as limited digital literacy and restricted access to technology can further complicate the use of mobile apps by older adults [28,29].

We recognize that the successful integration of technology into the lives of older adults requires careful consideration of usability, accessibility, and content relevance. In response to these multifaceted challenges, we adopted an approach that aimed to simplify the user experience by designing an app featuring simplified interfaces, cleared navigation, preloaded short video contents, and offline functionality to cater to the diverse needs and abilities of older users. In collaboration with the Portuguese Directorate-General for Health (DGS), our research group developed a free educational mobile app, called DigiAdherence, to increase health knowledge and prevent the most frequent health conditions among older adults, such as nutritional problems, physical inactivity, falls, polypharmacy, treatment adherence, and cognitive decline [30]. Our emphasis was on improving usability, ensuring the app’s simplicity, intuitiveness of use, and pleasant aesthetics, while also enhancing its utility for knowledge acquisition and application. By offering a straightforward approach to accessing health-related content, we aimed at minimizing barriers to use and promoting the engagement of older adults with digital health content. Importantly, this pilot intervention was conducted as a small-scale test to gather insights and feedback from users. Our goal was not to compete with the extensive pool of existing apps but to offer a preliminary or initial solution while aiming to refine and improve our approach based on the responses of this specific user group.

### Objectives

The primary aim of this study was to assess the usability and utility of the DigiAdherence app, specifically among an older adult population (65 years or older), during 30 days of use of its health-informative video-based content. The secondary aim was to assess behavioral change or knowledge improvement regarding treatment adherence, fall prevention, and health-related QoL 60 days after the intervention.

## Methods

### Study Design

This pilot study used a noncontrolled quasi-experimental design with outcomes measured at 2 assessment moments (first and second assessments). The usability and utility of the DigiAdherence app were evaluated 30 days after participants started using it (first assessment) and 60 days after they stopped using it (second assessment). All eligible participants were patients at the primary health care center of Portimão, Barlavento Primary Healthcare Centres (ACES Barlavento), Algarve, Portugal.

After being recruited and consenting to participation, participants attended 3 evaluation moments: 1 baseline assessment and 2 follow-up assessments (first and second assessments). The same investigator carried out all assessments and data collection.

The baseline assessment was performed before participants started using the app, between May and June 2021. Each visit had a duration of 30 to 40 minutes, during which participants were assisted in answering a questionnaire, and the mobile app was installed. If the installation was successful, participants were given a demonstration of the app’s functioning and encouraged to access its content themselves by selecting videos, adjusting the sound volume, changing the view to full-screen mode, and going back to the video selection process so any questions could be clarified. Access to the app was granted for 30 days, during which the user could easily access it offline and use it multiple times and at any time. The follow-up assessments were performed through phone call interviews 30 days after participants started using the app, between June and August 2021 (first assessment), and 60 days after participants stopped using it, between August and October 2021 (second assessment). At the end of the first assessment, participants were given instructions to uninstall the app or not to use it until the next assessment. Each follow-up assessment had a duration of 10 to 15 minutes, during which participants were assisted in answering a questionnaire, and the outcomes were assessed.

### Study Population

We used a convenience sample of 26 older adults 65 years or older.

#### Inclusion and Exclusion Criteria

Individuals were included if they (1) were 65 years or older, (2) were living at home, (3) owned an Android smartphone or a tablet, (4) were a Portuguese speaker or understood the Portuguese language, (5) were a patient in the primary health care center of Portimão, and (6) were willing to participate and gave written informed consent.

Individuals were excluded if they (1) did not know how to use a smartphone or tablet, (2) owned a smartphone or tablet with an operating system other than Android, (3) were unable to complete app installation, (4) did not use the app during follow-up, and (5) had any cognitive impairment or were unable to answer the questionnaire, assessed by the physician.

### Intervention (DigiAdherence)

**Figure 1:**
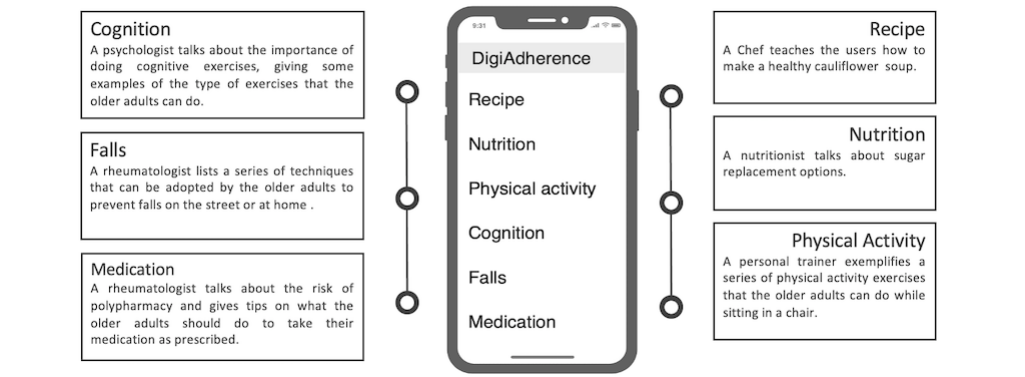
Depiction of DigiAdherence’s menu for accessing its health-informative video-based content.

Our research group developed a simple, intuitive, video-based mobile app containing 6 short health-informative videos (Figure 1) as described elsewhere [24]. Going through DigiAdherence’s menu, the user could choose any of its contents, in no specific order, after 2 simple taps. The “Recipe” content showed a chef teaching users how to make a healthy cauliflower soup; the “Nutrition” content showed a nutritionist talking about sugar replacement options; the “Physical activity” content showed a personal trainer exemplifying a series of physical activity exercises that users could do while sitting in a chair; the “Cognition” content showed a psychologist talking about the importance of cognitive exercises and giving some examples of the type of exercises that users could do; the “Falls” content showed a rheumatologist listing a series of techniques that users could adopt to prevent falls on the street or at home; and the “Medication” content showed a rheumatologist talking about the risk of polypharmacy and giving tips on what users should do to take their medication as prescribed. Every professional made sure to speak clearly without using medical or technical jargon, keeping their messages simple and easy to understand. Videos were stored locally within the app, enabling offline functionality, and each had a duration of 2 to 8 minutes. The executable file (APK) was created using Android Studio’s software. The app was installed on the participant’s own smartphone or tablet by one of the investigators during a scheduled in-person visit. After installation, participants had free access to the app for 30 days. More details on the DigiAdherence app can be found in the study protocol published elsewhere [30].

### Outcomes—Definition and Assessment

#### Overview

The primary outcomes were the DigiAdherence app’s usability and utility. The secondary outcomes were behavioral change or knowledge improvement regarding treatment adherence and health perception 60 days after the intervention.

#### Usability and Utility (Primary Outcomes)

The usability and utility of the app were assessed during the first assessment, whereas during the second assessment, only the utility of the app based on the knowledge acquired through it was measured.

First, participants were asked whether they use the app, with the question: “Have you used the app on your smartphone or tablet?” The first assessment was based on 8 items (5 instrumental items and 3 noninstrumental items) using a 7-point Likert scale. The 3 noninstrumental items assessed were the app’s visual aesthetics (“How do you classify the app in terms of pleasantness of its visual aesthetics?”), motivation to use the app (“How do you classify your motivation to use the app?”), and overall satisfaction with the app (“How do you classify your overall satisfaction with the app?”). The 5 instrumental items assessed were utility in taking medication correctly (“How do you classify the app in terms of usefulness to take your medication correctly?”), utility in QoL improvement (“How do you classify the app in terms of usefulness to improve your quality of life?”), utility in increasing health knowledge (“How do you classify the app in terms of usefulness to increase your health knowledge?”), utility in preventing falls (“How do you classify the app in terms of usefulness to prevent falls”), and the app’s easiness of use (“How do you classify the app in terms of easiness of use?”). Sixty days after the intervention (second assessment), participants were asked if they used knowledge acquired through the app to prevent falls (“Did you use the knowledge acquired through the app in terms of techniques for fall prevention?”—yes/no) and take their medication correctly (“Did you use the knowledge acquired through the app in terms of techniques for taking your medication correctly?”—yes/no). As an offline app, we did not quantify the app’s engagement metrics, such as frequency of use, exit rate, or use rate.

#### Treatment Adherence and Health-Related QoL (Secondary Outcomes)

Data measurement scales were used in all assessments to assess participants’ QoL (using the Portuguese version of the EQ-5D-3L questionnaire [31,32]) and treatment adherence (using the Portuguese version of the Medication Adherence Rating Scale [33]).

The EQ-5D-3L questionnaire includes 5 questions that describe health status in 5 domains: mobility, self-care, usual activities, pain and discomfort, and anxiety and depression. Each dimension had 3 levels: without problems, some problems, and extreme problems, and participants were asked to mark the option that would best describe their situation on that day. A preference-based scoring function was used to convert the descriptive system to a summary index score ranging from 1 (full health) to 0 (the worst possible state of health). Scores could also be negative down to –1, corresponding to death or states worse than death. Participants were also asked to assess their general health status on a visual analog scale from 0 (worst imaginable health status) to 100 (best imaginable health status), often called the EQ-VAS thermometer [31,32].

The Medication Adherence Rating Scale is a 10-item yes/no self-report instrument that was developed from 2 existing scales, the 30-item Drug Attitudes Inventory [34] and the 4-item Medication Adherence Questionnaire [35], to develop a more reliable and valid tool for assessing medication adherence behavior in psychosis. Total scores range from 0 (low likelihood of medication adherence) to 10 (high likelihood of medication adherence) [33].

#### Other Assessments

Information on sociodemographic data, such as sex, age, and years of education, was collected at the baseline assessment. Health literacy on fall prevention was also assessed (“Does the participant consider himself or herself familiar with fall prevention techniques?”—yes/no). When the answer was “yes,” the participant was asked to describe at least 1 strategy. The following parameters (except the app’s usability and utility, which were assessed during the follow-up assessments) were measured in the 3 assessments. Self-reported height and weight were measured, and BMI was calculated and categorized according to the World Health Organization classification (underweight: <18.5 kg/m^2^, normal weight: 18.5–24.9 kg/m^2^, overweight: 25–29.9 kg/m^2^, or obese: ≥30 kg/m^2^). Questions concerning clinical history included self-reported comorbidities (yes/no and comorbidity count), concomitant medication (yes/no and number of medications), and frequency of falls in the previous month (yes/no and number of falls).

### Statistical Analysis

A descriptive analysis of sociodemographic and clinical characteristics was conducted for the study population using frequencies and proportions for categorical variables and mean and SD for continuous variables. The app’s usability and utility are reported as median and IQR. Wilcoxon signed rank tests were used to compare treatment adherence perception and health perception between the baseline and first assessments and between the baseline and second assessments. Hypothesis testing (H0: median of the difference between the baseline and first assessments is 0; H0: median of the differences between the baseline and second assessments is 0) considered only participants with complete EQ-5D-3L and the Medication Adherence Rating Scale scores for both intervals. The significance level was set at .05. All analyses were performed using Stata/IC (version 16.1; StataCorp).

### Ethical Considerations

This study was submitted and authorized by the Executive Board of ACeS Algarve II – Barlavento (No. 16/2020) and by the Ethics Committee of NOVA Medical School (99/2019/CEFCM, June 2020), NOVA University of Lisbon, Portugal. This protocol was also submitted to and approved by the Ethics Committee for Health (16/2020, September 2020) and the Executive Board of the Regional Health Administration of the Algarve (December 2020), IP (Instituto Público) (No. 16/2020). All procedures followed the principles of Good Clinical Practice and the Declaration of Helsinki (Fortaleza revision, 2013). Participants were included in the study only after obtaining their written informed consent. Although this pilot study was based on the use of a mobile phone app, this was an educational app that only delivered short videos with health-informative content. It should be noted that this technology did not collect or transmit any personal data related to participants’ health through communication networks. Thus, according to the General Regulation on Data Protection Regulation (European Union) 2016/679 of the European Parliament and the Council on April 27, 2016, and the Regulation No. 1/2018, this study was exempt from notification to the Portuguese Data Protection Authority (*Comissão Nacional de Proteção de Dados*).

## Results

### Baseline Characteristics

**Figure 2:**
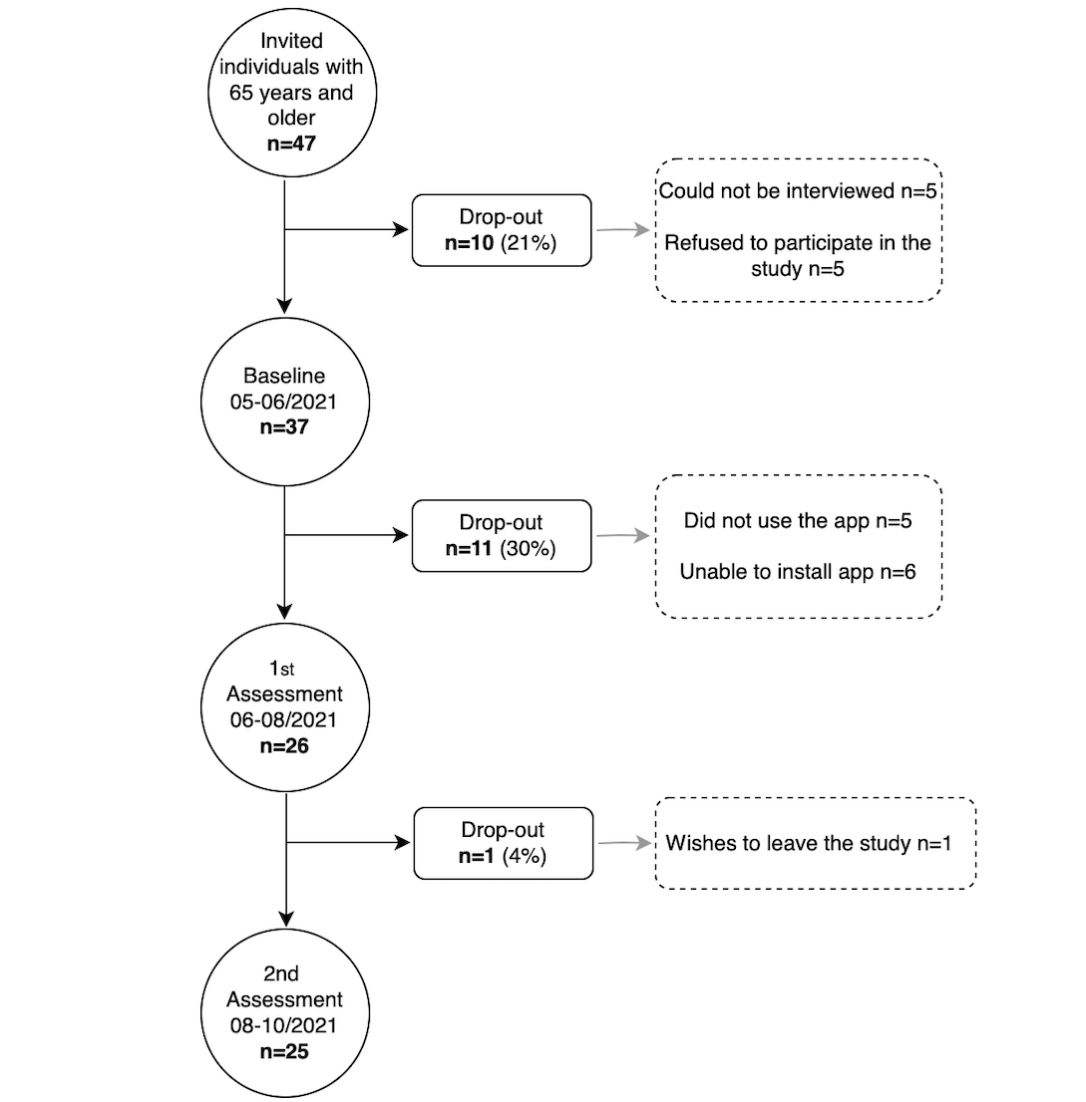
Flowchart of the DigiAdherence study.

Among a total of 47 older adults (23 male and 24 female) who initially showed interest in participating in this study and met the inclusion criteria, 37 agreed to attend an in-person visit at the primary health care center of Portimão. Of these older adults, 6 (16%) were unable to install the app because of insufficient storage space or the operating system not being compatible, and 5 (13%) installed the app but did not use it (Figure 2). Table 1 presents the sociodemographic and clinical characteristics of participants at baseline who successfully installed the app and used it for 30 days (26/37, 70%). The number of men and women were similar, with a mean (SD) age of 71.7 (5.4) years. The most common level of education was the 12th grade (9/26, 35%). Most participants were overweight (11/26, 42%). All participants had at least 1 comorbidity, with a mean (SD) of 3.6 (1.6) comorbidities. Of the 26 participants, 25 (96%) were on chronic medication. At baseline, 4 (15%) participants reported having fall prevention literacy, and 1 (4%) participant had experienced a fall in the previous month.

**Table 1:** Sociodemographic and clinical characteristics of participants at baseline who successfully installed the app and used it for 30 days (N=26).

Characteristics		Values
Women, n (%)		13 (50)
**Age (years)**		
	Mean (SD)	71.7 (5.4)
	65-69, n (%)	9 (35)
	70-74, n (%)	11 (42)
	≥75, n (%)	6 (23)
**Educational attainment, n (%)**		
	Basic education—1st cycle (4th grade)	5 (19)
	Basic education—2nd cycle (6th grade)	3 (12)
	Basic education—3rd cycle (9th grade)	5 (19)
	Secondary education (12th grade)	9 (35)
	Bachelor’s degree	4 (15)
**BMI (kg/m^2^), n (%)**		
	Normal weight (18.5-24.9)	8 (31)
	Overweight (25-29.9)	11 (42)
	Obese (≥30)	7 (27)
**Comorbidities**		
	Presence of comorbidities, n (%)	26 (100)
	Number of comorbidities, mean (SD)	3.6 (1.6)
**Chronic medication**		
	Chronic medication use^a^, n (%)	25 (96)
	Number of medications, mean (SD)	3.4 (1.8)
Fall prevention literacy (yes), n (%)		4 (15)
Falls in the past month (yes), n (%)		1 (4)
Health perception (0-100), mean (SD)		70.8 (16.5)
Quality of life (EQ-5D-3L scale), mean (SD)		0.70 (0.19)
Treatment adherence (MARS^b^), mean (SD)		8.1 (1.2)

^a^Sample size is not constant due to missing values for some variables: medication adherence (n=25).

^b^MARS: Medication Adherence Rating Scale.

Participants had a mean (SD) QoL score of 0.70 (0.19) and a mean (SD) treatment adherence score of 8.1 (1.2). Considering the mean scores for QoL and therapeutic adherence, it is evident that at baseline, the QoL is close to the national levels for the Portuguese population in the considered age group (0.790). Moreover, for therapeutic adherence, a value above 8 on a scale of 10 also indicates that the population already had good adherence at baseline.

The reasons reported by participants for not using the app were shortage of time and professional issues (n=2), depression (n=1), hospitalization (n=1), and no interest in the app (n=1). All participants had the app installed on their own smartphone (no tablets were used), except 4 who had it installed on their partner’s smartphone to avoid exclusion due to incomplete installation.

### Usability and Utility

**Figure 3:**
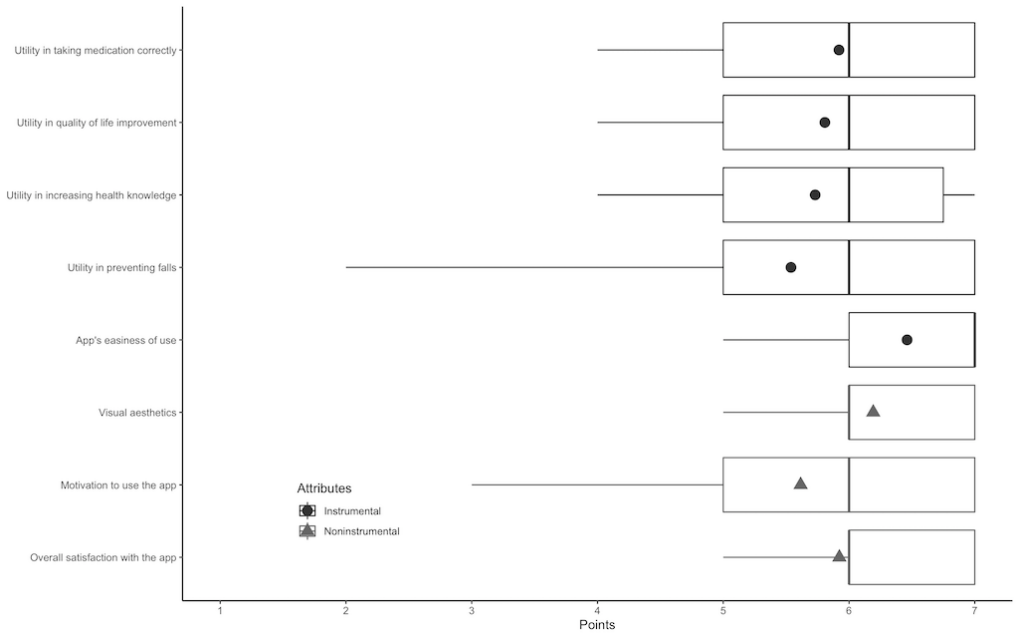
The usability and utility of instrumental and noninstrumental items measured 30 days after participants started using the app. The boxplots show the median (IQR, 25th and 75th percentile) and minimum and maximum points for each instrumental and noninstrumental item. The circles (instrumental items) and triangles (noninstrumental items) show the average score for each item.

All items had an average score above 5 and a median score of 6 on a 7-point Likert scale, with the exception of the app’s easiness of use, which had a median score of 7. Overall, the items with higher values were overall satisfaction with the app, visual aesthetics, and the app’s easiness of use (Figure 3).

### Treatment Adherence and Health-Related QoL

Regarding treatment adherence, 16 of the 26 (62%) participants applied the knowledge acquired through the app. For treatment adherence and health-related QoL, no significant differences were found between the baseline assessment and the first and second assessments (Table 2).

**Table 2:** Health perception and treatment adherence in the first and second assessments versus the baseline assessment.

Outcome^a^	Baseline assessment	First assessment (30 days after participants started using the app)	*P* value^b^	Second assessment (60 days after participants stopped using the app)	*P* value^c^
Health-related QoL^d^ (EQ-VAS^e^), mean (SD)	70.8 (16.5)	68.8 (14.8)	.90	70.6 (13.7)	>.99
Treatment adherence (MARS^f^), mean (SD)	8.1 (1.1)	8.5 (0.8)	.46	8.6 (0.9)	.23

^a^Sample size is not equal between scales due to missing values for some variables: health-related QoL—second assessment (n=24); treatment adherence—baseline (n=25), first assessment (n=25), second assessment (n=23). Exact *P* values are displayed.

^b^Wilcoxon signed rank test: H0: median of the difference between the baseline and first assessments is 0.

^c^Wilcoxon signed rank test: H0: median of the differences between the baseline and second assessments is 0. Exact *P* values are displayed.

^d^QoL: quality of life.

^e^VAS: visual analog scale.

^f^MARS: Medication Adherence Rating Scale.

## Discussion

### Principal Findings

We developed the DigiAdherence mobile app with the primary aim of assessing its usability and utility among a study population of 26 older adults who used its health-informative video-based content for 30 days.

Most participants rated the different functionalities of the app (eg, utility in taking medication correctly, utility in QoL improvement, utility in increasing health knowledge, and utility in preventing falls) positively, with an average score of above 5 and a median score of 6 on a 7-point Likert scale. Participants reported overall satisfaction with the app and perceived it as useful, attractive, and user-friendly.

In addition to learning information, participants felt that the app’s use offered a sense of security and knowledge in preventing falls and managing therapy and polypharmacy. At baseline, only 4 of 37 (11%) participants reported having fall prevention literacy, and after the intervention, one-third reported using the knowledge acquired through the app regarding fall prevention techniques. Although all participants had at least 1 comorbidity and most were on chronic medication (25/26, 96%), no significant differences were found in health perception or treatment adherence after using the app, probably because these measures were already high at baseline. Furthermore, half of the study population had a high level of education (12th grade or bachelor’s degree). Nevertheless, after the intervention, 62% (16/26) of participants reported applying the knowledge acquired through the app regarding treatment adherence. Regarding QoL, the short follow-up period made its assessment not relevant.

### Comparison With Prior Work

In a review of mobile app–based health promotion programs by Lee et al [36], mobile app programs for the general population have mainly been used for weight control and physical activity. The most common goals of mobile app programs were to provide both health information and feedback (6/12, 50% of studies), followed by only providing feedback (3/12, 25% of studies) or information (2/12, 17% of studies) [36].

In another review of health promotion and disease prevention for older adults by Chiu et al [37], the most common intervention method was health promotion, with 322 of the 486 studies (66%) classified as evaluating health promotion interventions. Meanwhile, 264 (54%) studies were classified as evaluating screening, 114 (23%) as evaluating primary prevention, and 72 (15%) as evaluating social support [37]. The most common target was “disease-oriented” (214/486, 44% of studies), followed by “physical activity” (120/486, 24.7%), “general health” (118/486, 24.2%), “quality of life” (106/486, 21.8%), “cognitive function” (100/486, 20.5%), “frailty” (85/486, 17.5%), “nutrition” (72/486, 14.8%), “mental health” (57/486, 11.7%), “psychosocial functioning” (51/486, 10.5%), “independence” (26/486, 5.4%), “sleep quality” (13/486, 2.7%), and, finally, “addiction”(4/486, 0.8%) and “disability” (4/486, 0.8%). Concerning eHealth technology apps from 2015 to 2019, only 12 of the 486 studies evaluated the use of eHealth technology. These tools included virtual reality (n=3), smart homes and home health monitoring technologies (n=1), socially assistive robots (n=5), and electronic assistive technology (n=3).

Finally, in a scoping review, Wilson et al [38] evaluated barriers to and facilitators of the use of eHealth by older adults as reported in 14 papers. According to Wilson et al [38], the most prevalent barriers to eHealth engagement were a lack of self-efficacy, knowledge, support, functionality, and information provision about the benefits of eHealth for older adults. Key facilitators were active engagement of the target end users in the design and delivery of eHealth programs, support for overcoming privacy concerns and enhancing self-efficacy in the use of technology, and integration of eHealth programs across health services to accommodate commonplace multimorbidity.

Overall, our findings suggest that designing simple apps specifically for older adults can motivate them to use them and perceive their usage as valuable. Providing more user-friendly systems and enhancing skills in using smartphones among this age group may contribute to older adults’ willingness to use mobile app–based interventions for self-health management. These results appear to be similar to those reported by Jo et al [12]. There are some apps for older adults that promote social interaction, health, and well-being [7-9,37]. We previously developed one such app (Saúde.Come Senior), which was shown to be feasible and acceptable by users [18,19]. Although the range of information and communication technology (ICT) products is expanding, more research on this topic is still needed. Future research should engage older adults in developing technology based on their needs. Furthermore, factors that influence older adults’ willingness to use ICT, such as mobile apps, should be assessed to ensure its successful and effective implementation [39].

### Strengths and Limitations

Several strengths of this study deserve to be mentioned. First, all assessments and data collection were conducted by the same investigator, which may have minimized interviewer bias. Second, developing a simple, intuitive app with offline access to its content after 2 simple taps may have increased participants’ technology adherence. This approach aimed to minimize the need for complex interactions and reduce the potential barriers associated with navigation. Moreover, DigiAdherence is an offline app with no interaction or dynamic elements, which was probably why no difficulties in participants engaging with the app were observed. While there may be other apps and studies targeting older adults with different features and functionalities, the simplicity and offline functionality of our app might have served as an advantage. It made the content more accessible, considering the diverse needs and limitations of older users. The local storage of videos was chosen to facilitate easy installation and usage, especially considering factors like limited data plans or technical proficiency. Third, conducting a feasibility assessment for the DigiAdherence app was deemed redundant. This decision was based on the fact that the DigiAdherence content, originating from the Saúde.Come Senior trial [17], has undergone extensive implementation, testing, and validation, establishing its feasibility and acceptability among users. Besides, our primary focus centered on evaluating the usability and utility of the app, emphasizing its user-friendliness and effectiveness in meeting users’ health-related needs. By addressing these factors, we aim to contribute to the development of effective digital tools that meet the needs and expectations of older adults. Moreover, we ensured that the concepts of usability and utility were aligned with accepted definitions and frameworks within the field of mHealth app usability assessment [40]. We drew on established definitions and guidelines to shape our evaluation criteria [40]. This reference provided us with a solid foundation for assessing the usability and utility of the DigiAdherence app and ensured that our evaluation was consistent with accepted standards in the field. Lastly, the first and second assessments were performed through a phone call interview, which may have limited the number of participants lost during follow-up.

Despite these strengths, this pilot study has limitations that need to be addressed in future trials. First, the use of a convenience sample of older adults with higher digital literacy and access to ICT may have led to selection bias. In addition, being an offline video–based app increased the need for free storage capacity. The app being exclusive to Android was also a limitation. In some instances, it was necessary to erase some of the participants’ device content. Nevertheless, 6 participants were excluded because of insufficient storage space (or the operating system not being compatible), and 4 had the app installed on their partner’s device to avoid exclusion due to incomplete installation. These participants were not excluded because we were assured that this procedure would not interfere with their access to the app. Regarding the sample size, it is important to clarify that our study’s primary objective was to assess usability and utility and gather preliminary feedback. This approach is consistent with the goals of other studies, where smaller sample sizes were valuable in providing early insights into user experiences [41,42]. Our intention was not to offer a definitive assessment of the app’s effectiveness for all potential users. Instead, it was a critical first step in our iterative process of refining and enhancing the app’s design, with a broader adoption and improved service in mind. Second, the first assessment occurred during summertime, between June and August, which made it difficult to establish contact with participants, probably due to a lack of motivation or availability to participate during their vacations. As a result, all phone calls were made more than 30 days after participants started using the app (mean 45.4, SD 10.2, range 34-69 days). The second assessment occurred approximately 60 days after participants stopped using the app (mean 63.5, SD 8.4, range 41-75 days). Between these 2 assessments, some participants chose not to uninstall the app, with the condition that they would not use it until the study concluded. Third, when asked to score the app’s usability and utility, some users rated it considering personal gain, whereas others considered a perception of collective gain, such as “for me it was not that useful, since I was already familiarized with most of the app’s contents, but for other people I believe it can be very useful.” A few users expressed some discontent with the fact that the video content was always the same, which may have led to a loss of motivation for its use. Lastly, regarding the use of a 7-point Likert scale, users had an average score of above 5 and a median score of 6. This may be due to overall satisfaction with the DigiAdherence app, but considering the age group in question, we should question the possibility that the use of a 7-point Likert scale may have been too complex (numerical/noncategorical, with a wide range of choices) combined with the fact that the follow-up assessments were conducted through a phone call (which made it impossible for participants to visualize the scale).

### Conclusions

In conclusion, the findings of this pilot study show the easiness of use and aesthetic appeal of the DigiAdherence app, thus resulting in higher overall acceptance and satisfaction among an older adult population. Lessons on the usability and utility of this app can be used for other apps targeted at older adults. Moreover, this intervention program seems to have increased older adults’ security and confidence regarding health-related knowledge, particularly in preventing falls and managing therapy and polypharmacy.

## Abbreviations

ACES: Primary Healthcare Centres (in Portuguese, Agrupamento de Centros de Saúde)
ARS: Regional Health Administration (in Portuguese, Administração Regional de Saúde)
DGS: Portuguese Directorate-General for Health (in Portuguese, Direção-Geral da Saúde)
FEDER: Fundo Europeu de Desenvolvimento Regional
ICT: information and communication technology
mHealth: mobile health
POCTEP: Operational Programme for Cross-Border Cooperation Spain-Portugal (in Portuguese, Programa Operativo de Cooperación Transfronteriza España-Portugal)
QoL: quality of life

## Data Availability

The data sets used or analyzed during this current study are available from the corresponding author upon reasonable request.

## References

[1] (2022). Ageing and health. World Health Organization.

[2] Population structure and ageing. eurostat, Statistics Explained.

[3] (2011). mHealth: new horizons for health through mobile technologies: second global survey on eHealth. World Health Organization.

[4] Arriaga M, Francisco R, Nogueira P, Oliveira J, Silva C, Câmara G, Sørensen K, Dietscher C, Costa A (2022). Health literacy in Portugal: results of the health literacy population survey project 2019-2021. Int J Environ Res Public Health.

[5] Koh A, Swanepoel DW, Ling A, Ho BL, Tan SY, Lim J (2021). Digital health promotion: promise and peril. Health Promot Int.

[6] Instituto Nacional de Estatística.

[7] Mira JJ, Navarro I, Botella F, Borrás F, Nuño-Solinís R, Orozco D, Iglesias-Alonso F, Pérez-Pérez P, Lorenzo S, Toro N (2014). A Spanish pillbox app for elderly patients taking multiple medications: randomized controlled trial. J Med Internet Res.

[8] Zhou L, Bao J, Setiawan IMA, Saptono A, Parmanto B (2019). The mHealth App Usability Questionnaire (MAUQ): development and validation study. JMIR Mhealth Uhealth.

[9] Changizi M, Kaveh MH (2017). Effectiveness of the mHealth technology in improvement of healthy behaviors in an elderly population-a systematic review. Mhealth.

[10] Oh YS, Choi EY, Kim YS (2018). Predictors of smartphone uses for health information seeking in the Korean elderly. Soc Work Public Health.

[11] Rasche P, Wille M, Bröhl C, Theis S, Schäfer K, Knobe M, Mertens A (2018). Prevalence of health app use among older adults in Germany: national survey. JMIR Mhealth Uhealth.

[12] Jo HS, Hwang YS, Dronina Y (2021). Mediating effects of smartphone utilization between attitude and willingness to use home-based healthcare ICT among older adults. Healthc Inform Res.

[13] Fairman AD, Dicianno BE, Datt N, Garver A, Parmanto B, McCue M (2013). Outcomes of clinicians, caregivers, family members and adults with spina bifida regarding receptivity to use of the iMHere mHealth solution to promote wellness. Int J Telerehabil.

[14] Parmanto B, Pramana G, Yu DX, Fairman AD, Dicianno BE, McCue MP (2013). iMHere: A novel mHealth system for supporting self-care in management of complex and chronic conditions. JMIR Mhealth Uhealth.

[15] Seto E, Leonard KJ, Cafazzo JA, Barnsley J, Masino C, Ross HJ (2012). Mobile phone-based telemonitoring for heart failure management: a randomized controlled trial. J Med Internet Res.

[16] Seto E, Leonard KJ, Cafazzo JA, Barnsley J, Masino C, Ross HJ (2012). Perceptions and experiences of heart failure patients and clinicians on the use of mobile phone-based telemonitoring. J Med Internet Res.

[17] Henshall C, Davey Z, Jacelon C, Martin C (2020). A usability study to test the effectiveness, efficiency and simplicity of a newly developed internet-based exercise-focused health app for lung cancer survivors (iEXHALE): protocol paper. Health Informatics J.

[18] Rodrigues AM, Gregório MJ, Gein P, Eusébio M, Santos MJ, de Sousa RD, Coelho PS, Mendes JM, Graça P, Oliveira P, Branco JC, Canhão H (2017). Home-based intervention program to reduce food insecurity in elderly populations using a TV app: study protocol of the randomized controlled trial saúde.Come senior. JMIR Res Protoc.

[19] Gomes LA, Gregório MJ, Iakovleva TA, de Sousa RD, Bessant J, Oliveira P, Branco JC, Canhão H, Rodrigues AM (2021). A home-based eHealth intervention for an older adult population with food insecurity: feasibility and acceptability study. J Med Internet Res.

[20] Ensuring Artificial Intelligence (AI) technologies for health benefit older people. World Health Organization.

[21] Nebeker C, Zlatar ZZ (2021). Learning from older adults to promote independent physical activity using mobile health (mHealth). Front Public Health.

[22] Cajita MI, Hodgson NA, Lam KW, Yoo S, Han HR (2018). Facilitators of and barriers to mHealth adoption in older adults with heart failure. Comput Inform Nurs.

[23] Terp R, Kayser L, Lindhardt T (2021). Older patients' competence, preferences, and attitudes toward digital technology use: explorative study. JMIR Hum Factors.

[24] To QG, Green C, Vandelanotte C (2021). Feasibility, usability, and effectiveness of a machine learning-based physical activity chatbot: quasi-experimental study. JMIR Mhealth Uhealth.

[25] Woods DL, Birren JE, Schaie KW, Satz (1991). The neuropsychology of aging. Handbook of the Psychology of Aging 3rd Edition.

[26] Melenhorst AS, Rogers WA, Caylor EC (2016). The use of communication technologies by older adults: exploring the benefits from the user's perspective. J Hum Factors Ergon Soc.

[27] Peek STM, Luijkx KG, Rijnaard MD, Nieboer ME, van der Voort CS, Aarts S, van Hoof J, Vrijhoef HJM, Wouters EJM (2016). Older adults' reasons for using technology while aging in place. Gerontology.

[28] Charness N, Boot WR (2009). Aging and information technology use. Curr Dir Psychol Sci.

[29] Xie B (2011). Effects of an eHealth literacy intervention for older adults. J Med Internet Res.

[30] Nunes-Da-Silva C, Victorino A, Lemos M, Porojan L, Costa A, Arriaga M, Gregório MJ, de Sousa RD, Rodrigues AM, Canhão H (2022). A video-based mobile app as a health literacy tool for older adults living at home: protocol for a utility study. JMIR Res Protoc.

[31] Ferreira LN, Ferreira PL, Pereira LN, Oppe M (2014). The valuation of the EQ-5D in Portugal. Qual Life Res.

[32] Ferreira LN, Ferreira PL, Pereira LN, Oppe M (2014). EQ-5D Portuguese population norms. Qual Life Res.

[33] Vanelli I, Chendo I, Gois C, Santos J, Levy P (2011). Adaptação e validação da versão portuguesa da escala de adesão à terapêutica [Medication adherence rating scale]. Acta Med Port.

[34] Hogan TP, Awad AG, Eastwood R (1983). A self-report scale predictive of drug compliance in schizophrenics: reliability and discriminative validity. Psychol Med.

[35] Morisky DE, Green LW, Levine DM (1986). Concurrent and predictive validity of a self-reported measure of medication adherence. Med Care.

[36] Lee M, Lee H, Kim Y, Kim J, Cho M, Jang J, Jang H (2018). Mobile app-based health promotion programs: a systematic review of the literature. Int J Environ Res Public Health.

[37] Chiu CJ, Hu JC, Lo YH, Chang EY (2020). Health promotion and disease prevention interventions for the elderly: a scoping review from 2015-2019. Int J Environ Res Public Health.

[38] Wilson J, Heinsch M, Betts D, Booth D, Kay-Lambkin F (2021). Barriers and facilitators to the use of e-health by older adults: a scoping review. BMC Public Health.

[39] Nordin S, Sturge J, Ayoub M, Jones A, McKee K, Dahlberg L, Meijering L, Elf M (2021). The role of Information and Communication Technology (ICT) for older adults' decision-making related to health, and health and social care services in daily life-a scoping review. Int J Environ Res Public Health.

[40] Venkatesh V, Bala H (2008). Technology acceptance model 3 and a research agenda on interventions. Decis Sci.

[41] Zapata BC, Fernández-Alemán JL, Idri A, Toval A (2015). Empirical studies on usability of mHealth apps: a systematic literature review. J Med Syst.

[42] Daniels J, Fels S, Kushniruk A, Lim J, Ansermino JM (2007). A framework for evaluating usability of clinical monitoring technology. J Clin Monit Comput.

